# Women’s healthcare needs and access across the incarceration pathway in Israel: a qualitative study

**DOI:** 10.1186/s12889-025-25389-y

**Published:** 2025-11-22

**Authors:** Nisreen Agbaria, Heino Stöver, Nadav Davidovitch, Shannon A. McMahon, Volker Winkler, Kate Bärnighausen

**Affiliations:** 1 Heidelberg Institute of Global Health (HIGH), Faculty of Medicine and University Hospital, Heidelberg University, Im Neuenheimer Feld 130.3, Heidelberg, 69120 Germany; 2 Department of Health and Social Work, Institute of Addiction Research (ISFF), Frankfurt University of Applied Sciences, Frankfurt am Main, Germany; 3 Faculty of Health Sciences, School of Public Health, Ben-Gurion University of the Negev, Be’er Sheva, Israel; 4 Department of International Health, Johns Hopkins Bloomberg School of Public Health, Baltimore, USA; 5 Wits School of Public Health, University of the Witwatersrand, Johannesburg, South Africa

**Keywords:** Incarceration, Women, Healthcare, Prison, Rehabilitation

## Abstract

**Background:**

Within punitive, under-resourced, and male-dominated incarceration systems, the healthcare needs of incarcerated women are often overlooked and inadequately addressed. This study aimed to explore the healthcare needs and access to care among formerly incarcerated women in Israel.

**Methods:**

We employed an exploratory-descriptive qualitative design, and interviews were conducted with eighteen formerly incarcerated women, purposively recruited from the Prisoner Rehabilitation Authority in Israel. Data were analyzed inductively using thematic analysis.

**Results:**

We group our findings into three overarching themes, representing the time-points illustrated by the participants: before incarceration, during incarceration, and after release from prison. Our themes illustrate the complex physical and mental health needs of formerly incarcerated women in Israel, perceived impact of incarceration on health, challenges faced by women with disabilities, discontinuity of care across stages of incarceration, mistrust in the prison medical system, systemic barriers to accessing timely and appropriate healthcare, and post-release support.

**Conclusions:**

This study emphasizes the urgent need for a trauma-informed, gender-responsive, and continuity-based approach to healthcare within carceral settings. Our findings support the need for integration of prison health services into the national health system and call for system-wide, cross-sectoral, and human rights-based policy frameworks that promote prevention, rehabilitation, and adequate mental and physical healthcare in prison. Integrating prison healthcare within the broader national health system is crucial for reducing health inequalities by improving continuity of care, transparency, and the effective sharing and reporting of health information.

**Supplementary Information:**

The online version contains supplementary material available at 10.1186/s12889-025-25389-y.

## Background

As of 2025, the global prison population is estimated to exceed 11 million individuals, including those held in pre-trial detention and those serving sentences [1]. Around 733,000 women and girls (including transgender women) are incarcerated worldwide, with significant regional variations, ranging from three per 100,000 population in Africa to 27 per 100,000 in the Americas [2]. Although women comprise a minority of the global prison population, their numbers have more than doubled over the past two decades, compared to a 22% increase among men during the same period, making them the fastest-growing segment of the prison population [2, 3]. This upward trend is largely driven by the criminalization of poverty, gender discrimination, structural inequalities, and harsh punitive policies for drug-related offences [4, 5]. Women in prison (WIP) are often detained for non-violent and less severe offences, stemming from social and health-related vulnerabilities, substance use (either dealing or using), and histories of physical and sexual abuse [3, 6, 7]. As proposed by Chesney-Lind (2006), women’s pathways into criminality are shaped by gender-specific risk factors that differ substantially from those influencing men [8]. These risks are compounded by intersecting social identities, socio-economic disadvantages, and histories of victimization [9]. Exposure to trauma and abuse is closely associated with mental health conditions, which frequently co-occur with substance use disorders (SUDs) [10]. Consequently, WIP experience substantial physical and mental health disparities compared to their male counterparts and women without incarceration histories [3]. These disparities extend to a wide range of physical conditions, including cardiovascular disease, cancer, and infectious diseases, and persist even after controlling for pre-incarceration socioeconomic factors [11–14]. Behavioural and mental health issues are consistently higher than among incarcerated men and women in the general population. These include SUDs, post-traumatic stress disorder, personality disorders, self-harm, and suicide [10, 15–17]. Maternal and reproductive health needs also remain largely unmet [18], and WIP often face increased risks for sexually transmitted infections and other reproductive health issues [19]. Incarcerated women frequently encounter barriers to reproductive care, limited access to hygiene products, and inadequate access to preventive screening [20]. Research further highlights experiences of physical restraints, lack of privacy, emotional neglect, and even violence during childbirth, all of which can lead to severe physical and psychological harm, and violation of basic rights [21, 22].

In Israel, around 190 women were incarcerated as of 2024, constituting about 2% of the total prison population [23]. These figures exclude Palestinian women, who are classified by Israeli authorities as ‘security prisoners’ [24]. The Israeli Prison Service (IPS) is the national authority responsible for managing the country’s prison system, operating 33 incarceration facilities, including nine detention centers [25]. Neve Tirza, established 1968 in the central town of Ramla, is Israel’s sole women’s prison. It houses both adults and minors incarcerated for a range of offences and sentence lengths [23]. Although many women are imprisoned for non-violent offences, the facility is designated as a maximum-security prison [26]. The facility consists of three main wings and sub-wings organized according to different criteria, including substance use treatment, and rehabilitation, mainly for women with addiction problems. The prison also contains two small factories (a sewing workshop and a carton factory) and an educational wing offerring vocational and leisure courses such as painting and drama. All women are required to either work or participate in educational activities during the day [26].

Israel’s National Health Insurance Law stipulates that Israeli residents are entitled to a set of healthcare services, provided by four non-profit health maintenance organizations [27]. However, this coverage explicitly excludes incarcerated individuals, and instead, their healthcare is operated by the IPS, separate from the Ministry of Health and without a clear policy framework for service organization [27, 28]. The IPS runs five medical centers within its facilities, staffed by around 43 doctors, only three of whom are specialists. The IPS health system does not include nurses in its staffing structure. Instead; some prison security staff receive basic first-aid training and serve as “medics” in shifts in the prisons’ clinics [29].

While lacking and inconsistently reported [30], available data shows disproportionately high physical and mental morbidity among incarcerated people in Israel compared to the general population. However, this data is not disaggregated by sex [31], leaving major gaps in understanding the healthcare needs of incarcerated women. A 2017 special report on incarcerated women in Israel reported that two thirds were mothers, and mostly single mothers of minors. They had higher rates of healthcare utilization, and about 40% were diagnosed with SUDs. The report also highlighted a shortage of mental health professionals at Neve Tirza prison [32]. Other studies present similar findings, indicating high prevalence of SUDs and severe psychiatric conditions, including suicidal ideation and suicide attempts [33]. Yet, the inadequate mental health staffing and lack of designated psychiatric hospitalization for women within the IPS poses significant challenges to providing appropriate mental healthcare [34].

During their imprisonment, some women undergo assessment to determine their eligibility for parole after serving two-thirds of their sentence, conditional on joining a rehabilitation program under the Prisoner Rehabilitation Authority (PRA). The PRA, a state authority operating under the Ministry of Welfare and Social Security in Israel, is tasked with supervision and design of community-based rehabilitation programs for individuals on parole or released after serving their sentences [35]. Eligibility assessment typically begins within three to six months before release. Those who join the rehabilitation program receive individual and family-based treatment, including advocacy, assistance in realizing and accessing social rights, and on-going guidance throughout re-entry and rehabilitation. Services are delivered through community-based centres and specialized facilities for individuals undergoing treatment for substance use. In 2023, 9,850 individuals were eligible to join the rehabilitation program. Of these men constituted the majority (97%), while women accounted for 3% [35]. At the time of study initiation, approximately 30 women were enrolled in PRA community-based programs.

## Methods

We employed exploratory-descriptive qualitative design (EDQ) [36] to explore women’s accounts of their healthcare needs and access to care during and after incarceration, including barriers, facilitators, and personal strategies for navigating the prison healthcare system in Israel. EDQ requires a flexible and inductive approach that captures the complexity and nuances of participant’s experiences [37, 38]. In this study, thirteen formerly-incarcerated women participated in semi-structured, face-to-face, in-depth interviews (IDIs), and five participated in a focus-group discussion (FGD).

### Participants and study setting

The PRA includes a ‘women unit’, which operates through different centers across Israel. All participants were recruited from three women-designated centres of the PRA, using a purposive sampling method to recruit adult (>18 years old) formerly-incarcerated women [39]. This study was initiated by the first author who contacted the PRA research unit and presented the intended study. Following the presentation, the study protocol was translated to Hebrew and was submitted to the research coordinator at the PRA along with an English version of the ethics approval. An online meeting was then conducted with the first author and senior members of the PRA research unit. During the meeting, technical and ethical aspects of the study were thoroughly discussed and clarified.

### Data collection

Following approval by the PRA, the first author, a female postgraduate researcher with experience in qualitative research and working with disadvantaged women across Israel, contacted the respective centers and scheduled visits. During these visits, the PRA staff at each center invited the participants to an introduction session, where they were introduced to the researcher. The lead author presented herself, her background, and the study’s aims both verbally and in written form. Participants had the opportunity to ask questions about the study, and some shared their experiences and thoughts on the topic. These conversations were not recorded, but participants were encouraged to revisit them during the interviews. After each introductory session, participants expressed their willingness to participate, or their decision to decline directly to either the researcher or the PRA staff. These preferences were discussed between the researcher and the staff, after which interviews were arranged. Written and verbal informed consents were obtained from those who wished to participate.

Interviews were conducted between February and April 2023. Interview guides were developed prior to data collection and further refined after the first two interviews to include additional prompts and questions on prison living conditions. Participants were asked to reflect on the experience of entering and leaving prison, the experience of seeking healthcare during and after incarceration, the living conditions in the prison, and the transition back to the community. Before the interviews, participants completed a socio-demographic questionnaire which included questions on demographics, incarceration history, and health status and behaviours. Interviews took place in private rooms without the presence of the PRA staff. The IDIs lasted between 20 to 75 min, while the FGD lasted 85 min. While we did not intend to conduct FGDs as part of data collection, we were advised via consultation with the PRA personnel that IDIs would not be appropriate due to time restraints within the participants’ obligatory schedule, and the preference of two participants who felt more comfortable speaking in a group with familiar and trusted peers. Therefore, at this particular centre we conducted one FGD and amended our guide to be appropriate for a group discussion. Interviews were conducted in the participants’ native language (Hebrew or Arabic) by the first author, a native Arabic speaker and fluent in Hebrew, which minimized the risk of linguistic misunderstandings. Notes were taken during the interviews, and all but two interviews were audio-recorded, transcribed verbatim, and proofed for accuracy. For the IDIs in which participants did not consent to recording, the first author documented key statements and information, and notes were added to the field diary.

Interviews were later translated into English by the first author as team members do not speak Hebrew or Arabic. Web-based translation tools like Google Translate were used to support the process, and all translations were checked and refined by the first author to ensure accuracy and preserve cultural and contextual meanings. Specifically, interviewer notes with cultural and contextual interpretations were added to the transcripts, drawing on her linguistic and cultural fluency in Hebrew, Arabic, and English. These notes were primarily intended to clarify religious or political aspects and events.

Follow-up and additional interviews were planned six months after the first round of data collection. However, this was not possible due to the security situation in Israel. By the 12th interview, we began observing a repetition of some themes, with core topics consistently recurring across interviews. Based on the quality of the interviews and richness of the information obtained, the sample characteristics and the preliminary analysis process, we felt that we gained sufficient information power and proceeded with formal data analysis [40].

### Procedures to ensure participants' wellbeing

All participants were given an information sheet which included a detailed description of the study aims and data collection procedures. During each round of data collection, PRA staff and the first author conducted debriefings to reflect on the research process and the sensitive nature of the topics discussed, while carefully considering participant’s reflections and emotional wellbeing. This preparation began prior to conducting the interviews through the preparatory meetings, which allowed women time to consider their participation in the study. This groundwork ensured that the study was situated aside the existing support structures at each PRA center. The researcher continuously emphasized that participants did not have to answer questions or disclose information if they were not comfortable to do so. Throughout data collection, iterative debriefing sessions were conducted with both the PRA staff and the participants to discuss the participants wellbeing, experiences, reactions, and feelings. If there were any issues, PRA staff were on hand to support the participant immediately.

### Study approval and ethical considerations

Ethical clearance was obtained from the Ethics Committee of the Medical Faculty of the University of Heidelberg (S-967/2021). The research was conducted in accordance with the principles outlined in the Declaration of Helsinki [41]. Particular attention was given to ensuring no harm to participants, maintaining anonymity and confidentiality, and upholding respect for participant’s rights, dignity, and welfare. Participants were compensated with an online voucher worth 50 Shekels (~$ 14 USD). The type and monetary value of the reimbursement were discussed and agreed upon with the PRA.

### Data analysis

Informal data analysis began during debriefing sessions [42]. Initial analysis was conducted by the first and last authors, and then shared and discussed with the rest of the authors. Guided by the six-step coding framework by Braun and Clarke [43], the first and last authors began with data familiarization, followed by inductive coding to generate initial codes that captured essential elements relevant to our research questions. We organized the codes into potential themes, where similar codes were grouped to form broader patterns. Finally, the process culminated in producing the first draft of the findings, where we used our core themes to build a coherent narrative. We embedded a reflexive process throughout, with team members continually discussing and reflecting on their own influence on the research, assumptions, and other personal, interpersonal and contextual factors [44]. Once the data had been translated to English, they were managed using NVivo-14 [45].

### Rigor and trustworthiness

Our team carefully navigated issues of credibility (confidence in the truth of the findings), transferability (the extent to which findings may apply to other contexts), dependability (consistency in the findings) and confirmability where findings are shaped by the participants’ accounts rather than researcher bias, to ensure rigor and trustworthiness in data collection and analysis [46, 47]. The first author conducted continuous debriefing sessions with the research team, where detailed description and reflexive thoughts were shared [44]. Reflexive notes captured the researcher’s own perceptions and cultural contexts in relation to the study. During the analysis process, the authors repeatedly revisited the original transcripts, observational notes and debriefing notes to ensure that our findings remained grounded in the data [42]. Additionally, the researcher addressed all questions related to future publication of the study and potential implications for policy and practice. After the preliminary data analysis, the first and third authors presented the findings to members of the PRA research unit. This study adheres to the consolidated criteria for reporting qualitative research (COREQ) checklist [48].

## Results

In total, 25 participants were approached, and twenty women agreed to participate in the study and completed the questionnaire. 

Eighteen participants then agreed to take part in interviews. Those who declined participation in the interviews cited distress related to their prison experiences. The median age of participants was 40 years, ranging from 22 to 60 years. Imprisonment duration varied widely, from four months to 16 years. For 70% of the participants, this was the first imprisonment. Although we did not have access to medical records, participants reported their history of disease through the questionnaire. Most reported that conditions were related to mental health, while one participant reported diabetes and another an undefined cardiovascular disease (Table 1). We group our findings into three overarching themes which represent the time-points illustrated by the participants: before incarceration, during incarceration, and after release from prison. Within these timeframes, we built sub-themes related to the disruption of care at the points of arrest and prison entry, living conditions in the prison, and barriers and facilitators to accessing healthcare services in prison. The themes are supported by quotations from the participants, identified by a letter and age-group to protect privacy.

**Table 1:** Summary of selected participants demographics and health characteristics

Variable	*n* = 20
Median age at the time of imprisonment	32
**Self-rated health status**	
Bad/Very bad	4 (20%)
Good/Fair	15 (75%)
Excellent	1 (5%)
**Commonly reported health conditions**	
Dental problems	13 (65%)
Migraine	13 (65%)
Chronic pain	12 (60%)
Depression/Anxiety	16 (80%)
Postpartum depression	3 (15%)
Post-Traumatic Stress Disorder	14 (70%)
**Does your health situation impact your daily function?**	
Most of the time	5 (25%)
Sometimes	12 (60%)
Little	3 (15%)
**History of abuse**	
Physical	8 (40%)
Sexual	8 (40%)
Psychological	9 (45%)
Experience of *≥* 2 forms of abuse	11 (55%)
**Marital status**	
Divorced	6 (30%)
Married	6 (30%)
Single	8 (40%)
**Education**	
High-School	2 (10%)
Vocational training	11 (55%)
Academic degree	2 (10%)
No certificate	5 (25%)
**Employment**	
Full-time	2 (10%)
Part-time	4 (20%)
Unable to work due to disability	4 (20%)
Not allowed to work	2 (10%)
Unemployed	8 (40%)

### Before incarceration: trial, detention and transition into prison

This theme illustrates the initial contact with the criminal justice system and the challenges women faced during this period. Women shared their experiences of being arrested and interrogated, as well as their experience during trial. Participants described feeling shocked and not being able to get prepared before the arrest. One participant, a woman with diabetes, was arrested at 04:00 a.m. and was not allowed to bring her medications:

*“I was in detention for 10 days before entering prison. I suffered from anxiety and stress during that period. I was arrested at 4 in the morning. I was in shock*,* it was traumatic… I fainted during the interrogation*,* and the doctor requested that I be transferred to the hospital*,* but that didn’t happen.” (L*,* 60)*.

In another case, a breastfeeding woman developed an infection due to her inability to nurse her child who was brought to the detention center by her mother every day. She later received a breast pump with the assistance of a social worker.

#### Psychological distress and disruption of care

Participants reported a range of health problems, primarily mental health issues experienced during legal proceedings, especially in cases of lengthy trial processes. They described significant anxiety and stress stemming from uncertainty during the trial period, which necessitated psychiatric and psychological therapy and affected their families as well, as trials could last for years:

*“The trial process lasted 4 years*,* during which I developed psychological illness. My husband had a heart attack during that time*,* I was the one who performed CPR on him. This made the anxieties worse. I started psychological treatment and started with medications.” (A*,* 50)*.

Others, knowing they would likely be incarcerated by the end of their trial, sought medical treatment in the community before entering prison.

#### Lack of mental health care during trial

Participants frequently cited a lack of understanding regarding their mental health conditions, particularly by the prosecution. They stated that these conditions were not considered during their trial and conviction. This was particularly evident in the accounts of two participants who reported suffering from postpartum depression during the time they committed their offenses:

*“They (the prosecution) were not able to appreciate or understand my situation that led me to do what I did*,* because they didn’t go through this experience of postpartum depression.” (F*,* 30)*.

One of them was also placed under psychiatric supervision before her sentence was issued:

*“The court decided that I will be transferred to a mental health facility for observation. There I was treated as a prisoner. I was there for a month and most of the time it was male police in the room*,* 90 shifts…sometimes*,* the officer would leave the cuffs on my hands even when I am in bed.” (S*,* 20)*.

#### Prison entry and discontinuity of care

 Prison entry was consistently described as a profoundly difficult and sometimes *“shocking”* and *“humiliating”* experience. Participants highlighted the drastic transition from being independent women with families to becoming part of a system where they were deprived from their freedom and autonomy.

*“I felt immense fear as I entered the prison*,* especially for a normative person like me*,* with family*,* grandchildren …At the beginning*,* I didn’t leave my cell for days. There was no explanation or guidance. No awareness. My anxieties got worse. How did I survive this? I don’t know.” (H*,* 60)*.

In some cases, women reported attempting to self-harm shortly after prison entry:

*“It was still difficult. I took it very hard. There were few times in the first days where I tried to harm myself.” (R*,* 50)*.

This transition also led to disruption in medical care, especially for women receiving psychiatric therapy or medications, and interruptions in follow-ups for treatments that had begun in the community. Some participants entered prison with their medical records; yet, they were not allowed to bring their own medications and sometimes they did not receive the same medications, but instead, they were prescribed substitutions by the prison doctor.

*“They (the prison clinic) provide alternatives and not the originally prescribed medications because some of the medications are not allowed in prison. I changed at least 4 times*,* some of these alternatives caused me severe depression.” (P*,* 40)*.

Nutritional supplements and vitamins were generally not provided, and women were not allowed to bring them into the prison either. One participant managed to get approval for vitamins through a court appeal, but she had to pay for them herself.

*“I told the prison staff that I needed vitamins. I was also depressed and I couldn’t eat. I told them what I used to take before prison*,* like b12*,* iron etc. But my requests were denied. I filed a legal petition*,* and after 4 months I got approval for multi-vitamin which I had to pay for myself.” (K*,* 50)*.

### During incarceration

#### Physical and personal boundaries in prison: overcrowding and shared spaces

Participants described the layout of the prison, highlighting its various wings and departments. They frequently described overcrowded cells, lack of proper ventilation, and sometimes lack of beds. While certain wings were designated for specific groups, such as for women with SUDs or mothers with their children, these designations were not always upheld. In some cases, women were placed in cells with others from vastly different backgrounds and offense categories, which some found distressing. For example, J., a 30-year-old participant incarcerated for a minor offense, was placed in a cell with two women convicted of violent crimes. She described how this impacted her:

“*I was in prison for a minor offence*,* I did not have problems before and I come from a good family*,* but being placed with women who committed violent offences was very difficult to me. I was exposed to their difficult life stories and circumstances*,* something I haven’t known before*,* and that was difficult.”*

Another participant recounted:

*“I was put in the same room with violent women*,* sex-workers*,* and others convicted of murder. It is not easy to get along. We all fight on the same territory.” (A*,* 50)*.

#### Nutrition, physical activity, and hygiene in prison

Prison conditions appeared to pose further challenges to maintaining health among incarcerated women. One participant described the prison as a *“very destructive place in terms of health”* (*Y, 30*). Others identified several factors that contributed to their ill health, including poor hygiene, low-quality drinking and washing water, rusty pipes and water taps, lack of exercise opportunities, and inadequate nutrition.

*“The nutrition is awful. It is mainly carbs*,* there is a lack of vegetables*,* and that’s why I avoided eating the prison food. I would only buy things from the canteen*,* but nothing really healthy…I even became vegan only to be able to get vegetables. We also experienced hair loss*,* infections*,* vomiting…” (Z*,* 30)*.

Exercise and sports were also limited. Participants reported a lack of space for physical activity, lack of structured sport classes, or inconsistencies in the scheduling of existing ones:

*“I continued to do sports as I used to do before prison*,* but it wasn’t always possible*,* they closed the courtyard*,* and there is no awareness of the importance of physical activity there…” (P*,* 40)*.

Additionally, lack of proper ventilation and lack of air conditioning in most of the cells, as well as extensive exposure to second-hand smoke were highlighted by the participants, both in the prison and detention centers. Exposure to smoke was reported in areas and cells that were designated as smoke-free. One participant, who complained about the heavy smoke exposure, said that she was left with long-lasting health problems:

*“I have a scar on my eye because of the exposure to smoke. Even in the therapeutic wing I was in*,* which is considered the best wing in the prison*,* everyone smokes. I appealed to the court*,* I won*,* but it didn’t help*,* they (the prison management) do not implement court orders.” (Y*,* 30)*.

#### Barriers for women with disabilities

Participants emphasized that the prison environment was not adequately equipped to accommodate people with disabilities. One participant reported that she had to negotiate for necessary accommodations both before and upon entering prison. Although she obtained a court order mandating these adaptations, she stated that they were only partially implemented by the IPS. Eventually, she was placed in a cell with other women who assisted her in navigating the new environment.

*“I have a disability*,* so in the beginning and despite my disability they put me in a cell*,* a smelly cell with four other girls. I didn’t know where to sit*,* I didn’t know what I’m allowed to sit on…No one provided me with an explanation or guidance. I cried and shouted*,* begged them to listen to me*,* but to no avail…” (Y*,* 30)*.

#### Emerging health issues during incarceration

Participants reported a wide array of physical and psychological conditions experienced during incarceration. Sometimes, these conditions existed before imprisonment but were exacerbated during incarceration, especially among women previously diagnosed with mental health conditions:

*“When I entered prison*,* I wasn’t able to function. I suffer from PTSD*,* bi-polar disorder and manic-depression. So*,* in such conditions*,* it exacerbates.” (S*,* 20)*.

Another participant added:

*“I suffered from migraine my entire life*,* and in prison it got worse. I also had sleeping problems so they also gave me a sleeping pill. Not that it helped…but better than nothing.” (P*,* 40)*.

One participant described having been successfully treated for a skin problem prior to incarceration. However, when the condition reemerged during her imprisonment, she reported not receiving appropriate treatment, ultimately resulting in disability. Others also cited physical conditions, including dental problems. Teeth pain and the need for dental treatments were common complaints, and were often met with sub-optimal and delayed care:

*“I had strong tooth pain in prison. I asked to see the dentist and he did some treatment. But they didn’t really treat me properly*,* they started telling me ‘we have a lot to do’… They gave me something for the pain*,* and I continued treatment after I was released.” (Z*,* 30)*.

Three participants with history of substance use described “forced abstinence”, where they were placed in isolation on their first days without access to professional or specialized treatment:

*“When I entered prison*,* I started asking for sleeping pills and I ate a lot*,* anything so I don’t think about drugs. I entered the prison 60 kg and left 95 kg after 8 months. I then decided with the PRA to get clean from drugs. They supported me with the process.” (D*,* 30)*.

#### Fragmented reproductive care

Participants reported fragmented or inaccessible gynecological services, as well as restricted and inconsistent availability of hygiene and sanitary products in the prison canteen. Two participants described entering prison during a critical time for their reproductive health: one shortly after childbirth, and another during follow-up for suspicious medical findings. Both reported extended delays in seeing a gynecologist, and one ultimately postponed the postnatal examination until after her release. Another participant reported that incarceration led to interruption in the fertility treatment she started prior to entering prison. On the other hand, participants recounted undergoing routine check-ups and screening services such as mammogram, provided by a local hospital, which were routinely scheduled on specific days.

#### Systemic barriers to healthcare in prison 

Women shared their experiences of seeking healthcare in prison, highlighting numerous challenges. They frequently described difficulties in securing medical appointments, as advanced registration was required and waiting times were excessively long. This was compounded by logistical constraints within the prison and by confusion among newly incarcerated women, as the procedures were unclear upon their arrival, as described by one participant:

*“To get a doctor appointment*,* you need to register a week before*,* but you don’t receive updates regarding your appointment request. The doctor arrives on certain days*,* and there is no guarantee that he will see you. Also*,* the movement in the prison is conditioned by accompanying guard. If there is no available guard*,* you will miss the doctor appointment. And then you need to wait another week at least.” (A*,* 50)*.

In most cases, medics were the first point of contact, but participants frequently described incompetency and lack of professional treatment. Some participants highlighted that prison doctors, who were often not specialized (i.e., only family doctors), provided sub-optimal care, and the waiting time to see a specialist was even longer.

*“A medic comes to the prison three times a day*,* and they are always there. But most of them lack professional training. There was a medic who didn’t know how to perform blood tests and also didn’t know how to read measurements. She told me that my blood pressure was 140 and asked if it was good… I replied*,* good for whom?” (Y*,* 30)*.

Others reported having to negotiate with the prison medical personnel and prison staff who discouraged them from pursuing certain examinations or treatments, particularly if they were nearing release, as one participant noted:

*“I went to the clinic for blood tests and the clinic director’s response was*,* ‘When are you supposed to leave prison?’ I asked why it was important for her to know*,* and she replied that she was just asking to know if it was worth it to do the tests now.” (S*,* 20)*.

#### Mistrust in the prison healthcare system

While some participants attributed the delay in medical appointments and treatments to limited sources and personnel within the prison, others described the advice as coming from a dismissive or judgmental attitude, and often felt that their health complaints were not taken seriously. Due to mistrust in prison healthcare personnel and the perceived low-quality of care, some women chose to delay treatment until their release:

*“I didn’t feel like I trust the medical staff there*,* and also*,* I didn’t feel like I wanted them to know my personal health issues. But then I decided to do it because I needed to take care of my health. But until this day*,* I didn’t get the results of my check-ups.” (B*,* 30)*.

#### Access to mental healthcare

The lack of specialized care extended to psychological services. Participants described long waits to see a psychiatrist, and when they did, these meetings were brief. Instead, social workers in prison were more frequently available, and experiences with social work programs were mostly described by the participants as positive:

*“For a long while after entering prison I was depressed and didn’t want to do anything. Then I met with a social worker who suggested that I take part in groups and such things. I started doing that and when my situation improved*,* I also started working in the prison. She supported me throughout the whole process.” (F*,* 20)*.

*“She (the psychiatrist) comes once a week to the prison*,* but you cannot register to get an appointment with her*,* but rather you have to tell your consultant and she calls her and tells her why you want to see her…and it doesn’t always happen” (S*,* 20)*.

#### Intersecting factors shaping access

Across the interviews, access to care and overall prison experience appeared to be shaped by intersecting factors, including socio-economic status, education, and social support. This was reflected in the ways some women were able to navigate the system to obtain healthcare services, which was possible as they had the required financial resources and support from outside the prison. For instance, some filed special petitions through their lawyers after being denied treatment by the prison or to request evaluation by a specialist:

*“I slipped on my back; I was taken to the hospital three days after my lawyer intervened.” (Y*,* 30) *.

*“Once you have a letter from your family doctor*,* and you don’t get a response from the prison*,* then you can use it to file petitions*,* then they (the IPS) have to do something about it. The prison services do not think out of the box*,* they never put any effort…” (P*,* 40)*.

Another participant recounted:

*“I moved between different wings. I was in the therapeutic wing for three years*,* which is the best. Really*,* you should just be a ‘positive inmate’. It is a wing for wealthy prisoners who are functioning in the prison and the management trusts them.” (Y*,* 30)*.

Although this issue was not commonly raised by the participants, especially among Arabic-speaking women who constituted less than half of the sample (8 out of 18), one participant explained that her limited Hebrew proficiency discouraged her from seeking medical care, especially as she was in mental distress during that time. Another participant stated that her Hebrew improved while in prison and that helped her manage her healthcare needs during and after incarceration.

#### Medical treatment outside the prison: shackling and lack of privacy

For participants who had to be transferred to an external healthcare facility (e.g., a public hospital), logistical and security considerations within the prison influenced the process. In some cases, women reported that transportation itself was inconvenient due to the shackling of their hands and feet during treatment as well, causing both physical discomfort and embarrassment about being handcuffed in public:

*“While I was in the hospital*,* I was in cuffs*,* on my hands and feet all the time. I told the guards it was painful and showed them the blue marks on my hands from the cuffs*,* but they refused to remove them. I went into the bathroom with one hand and one foot in cuffs.” (S*,* 20)*.

Lack of privacy was another challenge women faced during the hospital stay. They reported feeling uncomfortable by the constant presence of prison guards. Additionally, families were not notified in cases of hospitalization, and women were not allowed to contact them.

*“There are a lot of officers around you*,* sometimes including the intelligence officer… Even during the examination. When I had a gynaecological examination*,* they had to arrange a female guard to be me in the examination room” (F*,* 20)*.

#### Avoidance of medical care

In addition to the aforementioned factors that led women to avoid seeking care or delay it until after release, the challenges they faced while receiving medical care outside the prison led some to refuse or forgo medical appointments. This also occurred when women heard about experiences of others. The practice of being handcuffed, in particular, was a major deterrent:

*“It was very inconvenient to the point I gave up on many appointments. Even one time*,* after I had already arrived at the hospital. I entered prison shortly after I gave birth and I didn’t do any examination or follow-up*,* I just did them after my release to avoid the inconveniences and the humiliation” (F*,* 20)*.

On the other hand, participants noted that hospital staff treated them with sympathy. Some recalled instances where the staff members requested the removal of their handcuffs, but these were often denied by the guards who cited security concerns.

*“The medical staff at the hospital really do their best*,* if they see a male or female prisoner*,* I noticed that they try to shorten the waiting time as possible for them.” (F*,* 20)*.

#### Isolation and solitary confinement

Some participants reported experiencing various forms of solitary confinement, whether as punishment (e.g., after attempting self-harm), or due to being perceived as a threat to others. All women who experienced solitary confinement described it as a deeply distressing experience, with a long-lasting impact on their mental health. During the Covid-19 pandemic, isolation rooms were used for those who developed symptoms. As a result, some chose not to report symptoms to avoid being placed in isolation:

*“I was put in solitary confinement at 2 a.m.*,* in a cell we called ‘Halalit’- Hebrew for Spacecraft. I was terrified and begged the officer not to leave me there alone. She then put me in the ‘cameras room’. There is nothing there as well*,* but at least there is a guard and you can leave to work during the day… but still*,* this was more difficult than anything else I experienced in prison… I had psychiatric issues before that*,* and I was balanced with medications. But after solitary confinement*,* I started hearing voices in my head*,* something that I did not have before that.” (S*,* 20)*.

#### Lack of social support

Several women reported a lack of support from their families outside the prison. This affected the benefits they could have while in prison, such as receiving money transfers for the canteen or obtaining other items. In one case, a woman who was prescribed medication not provided by the prison could not get it as no one was available to bring it to her:

*“There is no one who can bring the medication to me from outside*,* so I waited until I was released and purchased it myself…” (A*,* 50)*.

Others described the methods and challenges of maintaining contact with their families. For those who had children, mothers or other family members cared for them while the women were in prison:

*“In prison*,* you get one video call for 15-30minutes once a month or twice a week*,* depending on the situation. On Mother’s Day we could see our kids for 3 hours. I had to go through a special committee first. I submitted the papers in August*,* and in November I received the response*,* close to my release day. The consultant told me: ‘you are leaving soon*,* why do you need that now!’. (S*,* 20)*

### Release from prison and transition into the community

#### Transition and fear of stigma

Women described their experiences of leaving prison and re-entering the community. While they expressed relief at regaining their freedom, many also faced difficulties adjusting. One participant described experiencing distress that led to physical health issues, as she recounted:

*“Upon my release*,* I started to have problems like vomiting and diarrhea*,* apparently my body was in stress this whole time. I took a one-week rest and then I went to the clinic to get treatment. Now I am waiting for my appointment with the psychiatrist to prescribe me new medications.” (P, 40)*.

Others reported mixed feelings during the transition period, particularly those with a history of family conflicts:

*“It gets more difficult with time. I don’t have patience anymore for my kids*,* so I reduced my time with them to twice a week. To get here (to the PRA centre) every morning and to deal with the situation is not easy*,* because here you have to face the things that happened and are still happening*,* so you sink…”* *(P, 40)*.

*“Of course*,* there is happiness*,* freedom has no price*,* but I returned to my family*,* who I have a lot of problems with*,* and traumas*,* nothing was fixed.”* *(S, 20)*.

Participants, mainly from the Arab-Palestinian community in Israel, expressed fears of stigma upon re-entering society. However, they did not experience discrimination from their doctors or healthcare providers:

*“People made comments and I could hear them saying ‘oh she was in prison’. Before*,* I would react immediately*,* constantly thinking and carrying it with me*,* but now*,* I don’t care.” (F*,* 20)*.

#### Re-establishing healthcare in the community

When asked about seeking healthcare and re-enrolling in the health insurance system, participants generally described a quick and smooth transition. The majority reported visiting their general practitioner at least once during the first month after release, and many expressed relief at being able to see their doctors again and receive care in a familiar setting. Most participants were unaware that their health insurance remained valid for the first year of imprisonment, and most also reported being unable to obtain their health records from the prison.

*“This is the most positive thing in the story. It was no problem to return. I was a bit shocked*,* finally sitting in front of normal doctors and speaking in a normal way… but like*,* it’s just an initial shock. The transition back into the healthcare system was quick and smooth.” (R*,* 50)*.

However, participants who needed continued psychiatric treatment after their release reported long waiting periods and difficulties securing appointments. Some struggled with scheduling conflicts, as their psychiatric treatment appointments overlapped with work or their mandatory program at the PRA:

*“I waited a very long time for a psychologist appointment. I was required to attend psychiatric treatment at a mental health facility*,* but the appointments clashed with my schedule at the PRA. Also*,* the therapeutic groups at that facility were not suitable for former prisoners.” (M*,* 60)*.

#### Social support and recovery

When asked about sources of support, the vast majority of participants identified the PRA and their relationships with social workers and staff members as central to their recovery and reintegration. These relationships were described as crucial in helping them rebuild their lives and regain a sense of stability and self-worth, as reflected in the interviews:

*“Right now*,* I am still in treatment here*,* so we continue the conversations. Otherwise*,* I would still be in prison… It helps the soul*,* and when the soul is healthy*,* the body is healthy too…” (B*,* 30)*.

*“I call her (the therapist in charge at the PRA)*,* and she always answers. Even on WhatsApp. She talks to me whenever I need to. The group meetings at the PRA helped me a lot. You talk*,* you ventilate. There are things the girls say that you can apply in your daily life.” (C*,* 20)*.

Another participant emphasized the impact of PRA's support in her life:

*“I wouldn’t be standing on my feet today without them. I’m clean from drugs and away from many bad influences. All of this is thanks to the individual and the group sessions and the support from the social workers here.” (F*,* 20)*.

This support was particularly important for women who had no prior access to psychological or social services, as stated by A., 60, who had spent over ten years in prison:

*“If I had the opportunity to talk and share what was going on in my life and receive proper help, I wouldn’t be sitting here today. I still come to the PRA, even after my mandatory period with them ended.”*

## Discussion

This study provides a deeper understanding of the healthcare experiences of formerly incarcerated women in Israel. Through thematic analysis, themes and subthemes were constructed from participants’ narratives and organized across three critical timeframes: pre-incarceration (trial and detention), during incarceration, and post-release. Across these time-frames, recurring themes present histories of trauma and their impact on physical and mental health [49], including the worsening of these conditions upon entering prison. The findings underscore systemic gaps in service delivery, including disruptions in medical care across stages of incarceration and failures to ensure continuity of care and information transfer during transitions. These shortcomings reflect organizational barriers to healthcare provision for incarcerated women in Israel, including lack of gender and trauma-responsive care, shortage of specialist doctors, long waiting times for appointments, and institutional factors that discouraged women from seeking care. Continuity of care is particularly critical for psychiatric conditions, where medications are often discontinued without appropriate clinical assessment in the early stages of incarceration [50, 51]. Individuals should be adequately informed about any medication changes, whether substitutions with different generic names or entirely new treatment.

Our analysis further emphasizes the role of these systemic factors in shaping healthcare access across the incarceration pathway, particularly the challenges stemming from the complete separation of prison healthcare services from the national health system and concerns regarding professional independence [27, 28]. This separation, combined with limited external oversight, places responsibility for the healthcare of one of the most marginalized populations in the hands of a security-oriented organization. The IPS is characterized by chronic underfunding, the absence of a coherent health policy, and inefficiencies in healthcare expenditures management [34].

Although research on this topic in Israel remains limited, previous studies have highlighted the physical and mental healthcare needs of incarcerated women [33, 52, 53]. Furthermore, reports from governmental bodies and human rights organizations have documented the unmet healthcare needs and gaps in provision within the IPS; however, these reports rarely include sex- or gender-disaggregated data [29, 31]. This gap became evident during the recruitment process, as both PRA staff and participants noted that, aside from research focused on specific issues (e.g., histories of abuse or substance use), this was the first study to examine women’s broader healthcare experience in this context. 

Our conceptualization of healthcare needs was grounded in the participants’ own narratives, in line with the EDQ design. This concept was built inductively as participants described their health and well-being priorities, including social, psychological, and physical needs, and experiences of accessing care during incarceration and reentry [54]. Women in our study demonstrated awareness of their healthcare needs and of the factors affecting their health and access to care. Moreover, participants cited issues of autonomy, privacy, and the frequent dismissal of their health concerns by the prison staff and medical personnel. These findings align with prior studies identifying barriers to prison healthcare, including limited accessibility, comprised confidentiality, the challenges of being recognized as legitimate patients, and conflicts between prison security policies and healthcare delivery [55, 56]. We also identified gaps in reproductive healthcare in the prison, highlighting neglect of gender-specific health needs. These gaps exacerbate vulnerabilities by delaying gynaecological evaluations or interrupting fertility treatments [57]. As Ross and Solinger [58] argue, reproductive justice frames access to fertility management, childbirth, and parenting as fundamental human rights, encompassing both the state’s duty to refrain from interference and its obligation to ensure equitable access. While debates persist about resources allocation for reproductive care in prisons, addressing existing gaps requires integrating appropriate reproductive healthcare into prison systems. This includes access to menstruation and hygiene products, timely prenatal and postpartum care [59], and continuity of care, while safeguarding the autonomy of women and their children.

Previous studies suggest that incarceration may sometimes offer positive health effects [60, 61], particularly through access to treatments previously inaccessible in the community (i.e., detoxification and psychosocial interventions) [62]. However, aside from occasional benefits, such as access to screening services, our findings highlight an overwhelmingly negative impact on women’s mental and physical health, which often persisted post-release, even with transitional support. Women attributed this to the perceived low quality of healthcare services in prison, mistrust in prison medical personnel, and judgmental or dismissive attitudes from the staff. Such experiences led some to delay seeking treatment until after release, compounding their health challenges. Participants also described how organizational and structural conditions in prison-limited diet diversity, restricted physical activity, and poor hygiene- impacted their health, self-esteem, and sense of worth [63]. Additional concerns included overcrowding, poor hygiene and water quality, and prolonged exposure to second-hand smoke, all of which were associated with heightened health risks in incarceration settings [64–66].

Some participants reported developing new health conditions shortly before or after entering prison, which they attributed to the intense stress associated with trial proceedings and the shock of imprisonment. This aligns with previous research identifying both acute and chronic stressors as mechanisms that negatively affect health during incarceration [67]. All women described experiencing what Sykes termed the “pains of imprisonment” [68]: humiliation, deprivation of liberty and privacy, separation from children and families, and loss of identity–as both human beings and women. While psychological distress upon prison entry may decline overtime as individuals adapt [65], our findings show that persistent stressors, both personal and institutional, continued to shape mental health outcomes. Participants reported diminished self-care capacity, erosion of privacy and dignity, and strained relationships with prison staff [69]. One major stressor was being housed with women with mental health conditions or histories of violence. Vaisman and Einat (2021) similarly found that such joint confinement caused significant distress for women without prior mental illness, potentially leading to long-term consequences. They recommended separating women with and without diagnosed mental illness, or ensuring appropriate support and rehabilitation [26]. In other cases, isolation or solitary confinement were described as one of the most distressing experiences, especially among women with pre-existing mental health issues [70, 71]. These factors further impacted mental and physical health, especially amid limited access to adequate healthcare [72]. Amid these challenges, the PRA plays a pivotal role in supporting women's re-entry. Participants described the PRA framework as a key source of emotional and practical support that helped them rebuild stability, maintain treatment continuity, and provide therapeutic framework, especially for women lacking prior psychological or social support. However, some participants reported scheduling conflicts between PRA activities and medical appointments. While the PRA is aware of these challenges, its operation is determined by legal requirements that limit flexibility. Furthermore, as mental health plays a crucial role in continuity of care within the PRA program, gaps in coordination place considerable strains on post-release support systems. These findings underscore the urgent need for a stronger integration between prison healthcare services and rehabilitation programs, and the implementation of effective medical information-sharing (i.e., interoperable electronic health records) to ensure continuity and quality of post-release care and reintegration [73]. Overall, the PRA's model demonstrates significant potential for facilitating reintegration and promoting well-being, warranting further exploration, as similar programs could strengthen continuity of care and social reintegration. 

Another concerning issue raised was the routine use of handcuffs and physical restraint during medical treatment. As visible in the findings, this practice extended beyond physical discomfort, often leading women to delay care to avoid being restrained. These findings are consistent with a retrospective study of 2,950 hospital visits by incarcerated individuals in Israel (2020–2022), which found that over 95% of patients were shackled during treatment, often with both hands and legs [74]. These findings emphasize the need for evidence-based, legally grounded guidelines that safeguard patients’ rights while balancing clinical and safety requirements [75]. Although participants in our study consistently described hospital staff as sympathetic, their accounts primarily focused on prison authority practices rather than provider interactions.

### Future considerations through an intersectional lens

While in this study intersectionality was not applied as a direct analytic framework, our findings indicate that incarceration pathways mirror and reinforce intersecting vulnerabilities that shape women’s health needs during and after prison. This perspective allows us to situate the complexity of healthcare access across different phases of incarceration [76, 77]. By considering intersectionality, we could highlight overlapping axes of vulnerability and privilege that influence women’s access to healthcare during incarceration [78, 79]. These include age, religion, ethnicity, gender imbalance within male-dominant system, prior trauma, disability, and socio-economic status. These intersections shape women’s ability to adapt even within controlled environments [80]. For instance, participants identified high socio-economic status, education, and family and social support as key factors enabling access to healthcare services that might otherwise have been unattainable. These women actively negotiated and leveraged the legal system and power dynamics within the prison to obtain needed healthcare [63]. 

While our study could not fully explore how certain axes interact, future work may benefit from a deeper investigation of how certain identities, such as nationality and ethnicity interact to affect or worsen physical and mental health of incarcerated women [77]. Few studies have examined intersectionality in prison health directly. Latham-Mintus et al., [81] found that incarceration history was associated with poorer health outcomes among older adults, especially older women of color. Another study linked life stressors to increased risky sexual behaviours among incarcerated cisgender Black gay and bisexual men compared to non-incarcerated men [82]. In the Israeli context, studies have shown that the intersection of race, ethnicity, geography, discrimination, immigration status, and socio-economic status shape health inequities, particularly among women [83, 84]. In conclusion, we posit that vulnerabilities intersecting with incarceration should be addressed earlier in the incarceration pathway, rather than only at its end. Early interventions are essential, especially for women who have experienced abuse and sexual trauma, an unaddressed gap, particularly in patriarchal and conservative societies. Applying this perspective proved valuable for foregrounding disparities, illness experience, and social justice through attention to structural influences in pursuing health equity, while acknowledging the methodological challenges of systematically operationalizing intersectionality in empirical research.

#### Limitations

The study recruited women from a single setting, the PRA, and our sample did not include other sub-groups like women who were with children in the prison or women in the substance use treatment programs within the PRA. Political prisoners were not included in this study as they are placed in separate detention centres and are subject to a different judicial system.

## Conclusion

This study presents insights into the healthcare needs of women who have experienced incarceration across the incarceration pathway in Israel. By centering the perspectives and experiences of formerly incarcerated women, this study highlights an often-overlooked population and provides critical insights into the challenges they face in accessing and receiving healthcare. We identify critical time-points where medical care interruption occurs and identify systemic factors contributing to these gaps. There is a need for developing context-specific models for integrating prison healthcare into the national health system, improving services delivery, and assessing the long-term health outcomes among incarcerated women. Finally, any future research and interventions should also consider the scale and effectiveness of alternatives to incarceration for women, especially those with psychological conditions. Future studies can benefit from improved data sharing and routine reporting practices that move beyond binary categorizations of incarceration and social factors such as sex/gender and ethnicity. Such advancements are essential for addressing the multifaceted health inequities faced by incarcerated women and for informing evidence-based interventions.

## Supplementary Information

Supplementary Material 1.

## Data Availability

The datasets generated and/or analyzed during the current study are not publicly available due to the inclusion of sensitive information and privacy protection reasons, but are available from the corresponding author on reasonable request.

## Abbreviations

WIP: Women in Prisons
SUDs: Substance Use Disorders
IPS: Israeli Prison Services
EDQ: Exploratory Descriptive Qualitative design
IDI: In Depth Interview
FGD: Focus Group Discussion
PRA: Prisoner Rehabilitation Authority
COREQ: Consolidated Criteria for Reporting Qualitative Research
